# β3-integrin Leu33Pro gain of function variant does not modulate inflammatory activity in human derived macrophages in diabetes

**DOI:** 10.7150/ijms.55648

**Published:** 2021-05-13

**Authors:** Philipp Helmer, Ellen Damm, Stephan Schiekofer, Kirsten Roomp, Jochen G. Schneider

**Affiliations:** 1 Saarland University, Medical Center, Dpt. of Internal Medicine II, Homburg, Saar, Germany.; 2 Zentrum für Altersmedizin, Klinik und Poliklinik für Psychiatrie und Psychotherapie der Universität Regensburg am Bezirksklinikum, Regensburg, Germany.; 3 Sigmund Freud Privat-Universität, Wien, Austria.; 4 Luxembourg Centre for Systems Biomedicine (LCSB), University of Luxembourg, Belvaux, Luxembourg, and Centre Hospitalier Emile Mayrisch, Esch/Alzette, Luxembourg.

**Keywords:** Leu33Pro polymorphism, rs5918, diabetes, diabetic nephropathy, inflammation, macrophages

## Abstract

**Objective**: We aimed to investigate the association between the Leu33Pro (rs5918) polymorphism in β3-integrin with diabetic complications and inflammatory function of macrophages depending on the genotype in subjects with diabetes mellitus.

**Material and methods:** We determined the Leu33Pro polymorphism in 186 diabetic subjects and collected laboratory data. Monocytes from 24 patients were collected for macrophage differentiation to determine the inflammatory activity by treating with different stimulants.

**Results:** We could demonstrate that human derived differentiated macrophages expressed β3‑integrin. Their secretory capacity upon inflammatory stimulation did not reveal any differences depending on the Leu33Pro variant. We found trends for an association of the polymorphism with the presence of diabetic nephropathy (*p = 0.071*), as well as with creatinine [1.32 mg/dL (1) vs. 0.98 mg/dL (0)] (*p = 0.029* in recessive model) and glomerular filtration rate [75.6 ml/min ± 22 vs. 62.3 ml/min ± 25] (*p = 0.076 in recessive model*) as quantitative markers of kidney function.

**Conclusion**: Despite the expression of β3‑integrin in human macrophages, the Leu33Pro polymorphism in β3‑integrin does not modify the inflammatory response upon stimulation but might play a role in the progression of diabetic nephropathy. Further studies are necessary to substantiate such a hypothesis.

## Introduction

ανβ3‑integrin (aka vitronectin receptor) is a heterodimeric cell surface receptor with varying functions depending on the level and location of its cellular expression. It is involved in signaling both between cells, and between cells and the extracellular matrix 1. ανβ3‑integrin ligation induces conformational changes that lead to the facilitation of signaling events within the cells with consequences such as migration, proliferation, and differentiation 2. Gene-knockout studies in mice suggested a role for ανβ3‑integrin in disease conditions such as inflammation, bone diseases and cancer 3-5. Consequently, β3‑integrin modulation, as a part of the vitronectin receptor, has been tested to reduce osteoporosis, tumor growth and angiogenesis by blocking β3‑integrin with antibodies or RGD peptide antagonists, yet the results are inconsistent. In clinical studies a natural modulation of β3‑integrin by the Leu33Pro polymorphism (rs5918) has been demonstrated to be associated with diabetes mellitus and furthermore diabetic vascular diseases 6, 7. The mechanism of this observation remains unclear so far. However, the Leu33Pro polymorphism results in a constitutively active β3‑integrin 8 and β3-integrin-deficient macrophages have been shown to be involved in inflammatory atherosclerosis 3.

The aim of our study was to investigate if there is an association between the Leu33Pro polymorphism with diabetic complications and to functionally characterize the inflammatory activity of human differentiated macrophages in carriers of the polymorphism versus controls.

## Material and Methods

The study was approved (11 June, 2015) by the ethics committee of Saarland (E242/14) and all participants gave written consent to the study (n=193). They were recruited from collaborating diabetic clinics and diagnose of diabetes was made according to American Diabetes Association criteria. 186 subjects (62% men, 38% females; mean age 64 years) were included into the study. Patient characteristics and biochemical parameters were determined from standardized clinical charts. The patients were genotyped for the Leu33Pro polymorphism using a Taqman assay modified from a previously published protocol 9. Blood was drawn from the study participants (n=24) for harvesting blood derived monocytes in Leucocept tubes (Greiner, Kremsmuenster, Austria) through Ficoll-Paque Premium density gradient centrifugation. CD14-expressing cells were selected using CD14+ micro-beads for incubation and magnetic activated cell sorting (MACS, Miltenyi biotech, Bergisch-Gladbach, Germany). The CD14+ cells were further differentiated into macrophages by incubation with X‑VIVO^TM^ 10 medium containing 100 ng/ml M-CSF for 8 days. The species and vitality of the cultured cells were confirmed by immunofluorescence. Macrophages were plated in equal numbers in 12 well plates and, after serum starvation, stimulated either with 100 ng/ml LPS, 200 nM PMA or 50 ng/ml interferon γ. MPO- and multiplex ELISAs were performed on cell supernatants according to manufacturer's instructions and normalized to cell protein concentration. Statistical methods comprised parametric and non-parametric correlation analyses, Chi-square test, ANOVA or non-parametric tests where appropriate. Statistical analyses were performed using SPSS, release 19 (IBM SPSS Statistics, Chicago).

## Results

**Figure 1:**
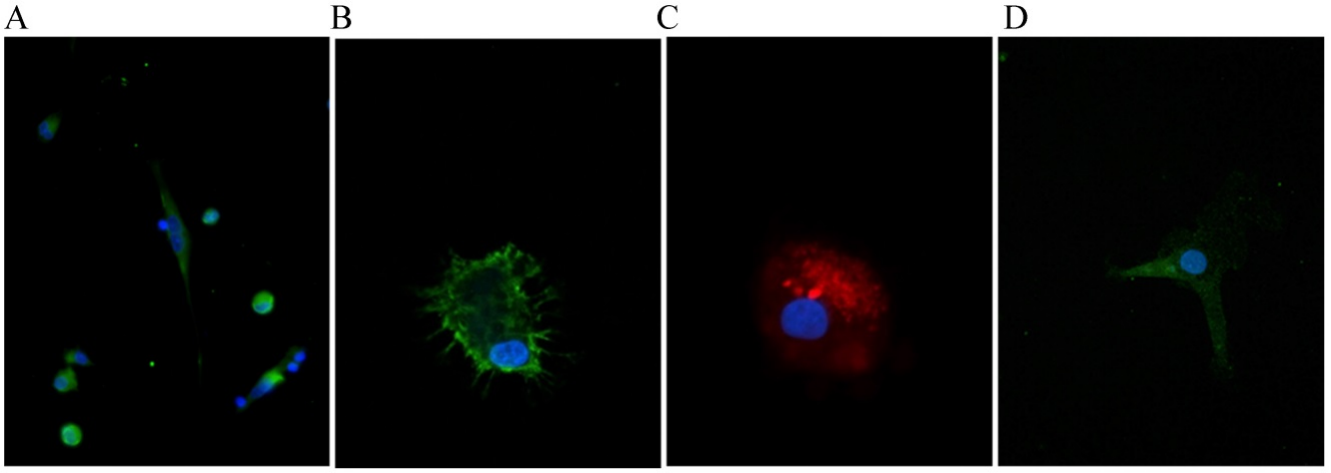
Representative immunofluorescent stain of macrophages for proof of appropriate differentiation: A and B. Stain for nuclei (blue, DAPI) and EGF-like module-containing mucin-like hormone receptor-like 1 (green, EMR-1). C. Nucleus (blue, DAPI) and Dil-ox-LDL 20 µg/ml). D. Nucleus (blue, DAPI) and β3-integrin (green). Imagination A: 600x, B-C: 1000 x.

The allele frequencies were 0.84 for the wildtype and 0.16 for the Leu33Pro polymorphism. The allele frequencies were in accordance to the Hardy-Weinberg-equilibrium (Chi-square *p=0.59*). The characteristics of the study population according to the genotype are displayed in **Table 1.** The cultivated monocytes from the diabetic patients with the different genotypes were differentiated into macrophages. Apart from morphology, we assessed the successful differentiation by verifying for expression of the macrophage specific marker EMR-1 and proper uptake of oxLDL by immunofluorescence to prove the vitality of the cultured cells (**Fig. 1A, B, C**). We could also show that β3‑integrin is expressed in unstimulated differentiated macrophages (**Fig. 1D**). We next examined the macrophage secretory capacity in response to the stimuli (LPS, PMA, Interferon γ) depending on the appropriate genotype. In this regard, we found no unifying and logical association of a genotype with the magnitude of any of the measured inflammatory markers.

Previous results suggested an association between the Leu33Pro variant in β3-integrin with macrovascular complications in diabetic patients 10. Here, we did not find a significant difference in the prevalence of diabetic micro- and macrovascular complications associated with the Leu33Pro polymorphism. We found that the relative frequency of diabetic nephropathy was 50% within the diabetic patients who were homozygous for the Leu33Pro polymorphism, 21% in diabetic patients homozygous for the wildtype, and 12% in the heterozygous diabetic patients. We also found indications of significant differences in GFR depending on the genotype in the overall ANOVA *(p=0.034)* accompanied by a corresponding trend (*p=0.112*) towards higher creatinine levels in the homozygous versus the wildtype group [1.32 mg/dL (0) vs. 0.97 mg/dL (0)].

Due to the overall low number of homozygous subjects in our study, we employed a recessive model to leave out one degree of freedom weight as compared to setting out 3 genotypes as separate categories. In this recessive model which requires homozygosity to show an effect we found a significant increase *(p=0.029)* of the creatinine levels in homozygosity (**Fig. 1**) as well as a trend towards reduced GFR rate (*p=0.076*) in the homozygous subjects as compared to the control group of heterozygous and wildtype patients.

## Conclusions

The exact pathophysiology of the diabetes complications remains incompletely understood. In addition to the toxicity of hyperglycemia, inflammatory processes also likely make an important contribution to the pathophysiology. Especially macrovascular complications appear to be a result of inflammatory vascular diseases and the β3-integrin has been implicated in inflammatory diseases and diabetes 3, 6, 11. These observations prompted us to examine macrophages from diabetic subjects carrying the Leu33Pro allele of β3-integrin. There are previous reports claiming that β3-integrin is not expressed in macrophages at all 12, and yet, we were able to show that β3-integrin is expressed in fully differentiated human macrophages **(Fig. 1).** To our surprise, we did not find a significantly and/or coherently altered inflammatory state in macrophages from subjects with the Leu33Pro variant of β3-integrin. Hence, the exact function of β3-integrin in macrophages remains to be explored as both pro-inflammatory 13 and anti-inflammatory roles are described 3 so far. We are tempted to speculate the Leu33Pro variant in β3-integrin does not modulate the macrophages and their innate immune activity, at least not in our cohort.

The present work demonstrated homozygosity for the Leu33Pro polymorphism in 3.23% and heterozygosity in 26.34% of the diabetic subjects and thus is in accordance to previously published literature 6. Reports on cohorts collected by other groups investigating different populations, show different allele frequencies, which suggests a significant dependence of the allele frequency on ethnicity 14.

We have found no association between the Leu33Pro variant in β3-integrin and the diabetic state or markers of metabolism, which is also in accordance to previously published literature 7. Our study was not designed and is too small to contribute to the large debate on the association of the Leu33Pro variant and coronary artery disease. However, we identified a trend towards the association of diabetic nephropathy and of its markers with theLeu33Pro allele in our small cohort. This finding is intriguing because β3-integrin has thought to play a role in diabetic kidney disease for years 15. Patients of our cohort showed a worsening of renal function with two mutated alleles of the polymorphism. Although we remain careful because of the size of our cohort and possible missed confounding factors, our results corroborate another study that showed podocyte damage in human diabetic nephropathy by activation of the β3‑integrin 16. The mechanism by which the activation of β3-integrin may lead to progression of diabetic kidney disease remains unclear. One could speculate that a constitutively active β3‑integrin may result in cytoskeletal re-organization leading to improper barrier function of the podocytes. Another consequence of this constitutively activate state could be a facilitation of neo-angiogenesis in the early phases of diabetic kidney disease. These speculations exceed the scope of the present work but are well corroborated by the finding that inhibition of ανβ3-integrin in pigs reduced diabetic nephropathy as compared to controls 14.

In conclusion, the Leu33Pro variant of the β3-integrin gene is associated with diabetic nephropathy suggesting that a constitutive active β3-integrin and not the magnitude of its expression may be associated with diabetic nephropathy by complex underlying mechanistic networks. The macrophages of the Leu33Pro diabetic subjects and their inflammatory capacity do not contribute to the explanation of the observed association. The missing underlying explanation warrants further basic research efforts and epidemiological evidence.

## Author Contributions

PH and ED performed the experiments and collected the data, KR and SS contributed to the data interpretation and helped writing the paper. JGS provided the study protocol and wrote the paper. PH and ED contributed to the study protocol and analyzed data. All authors interpreted the findings, edited and approved the final version of the manuscript.

## Figures and Tables

**Figure 2:**
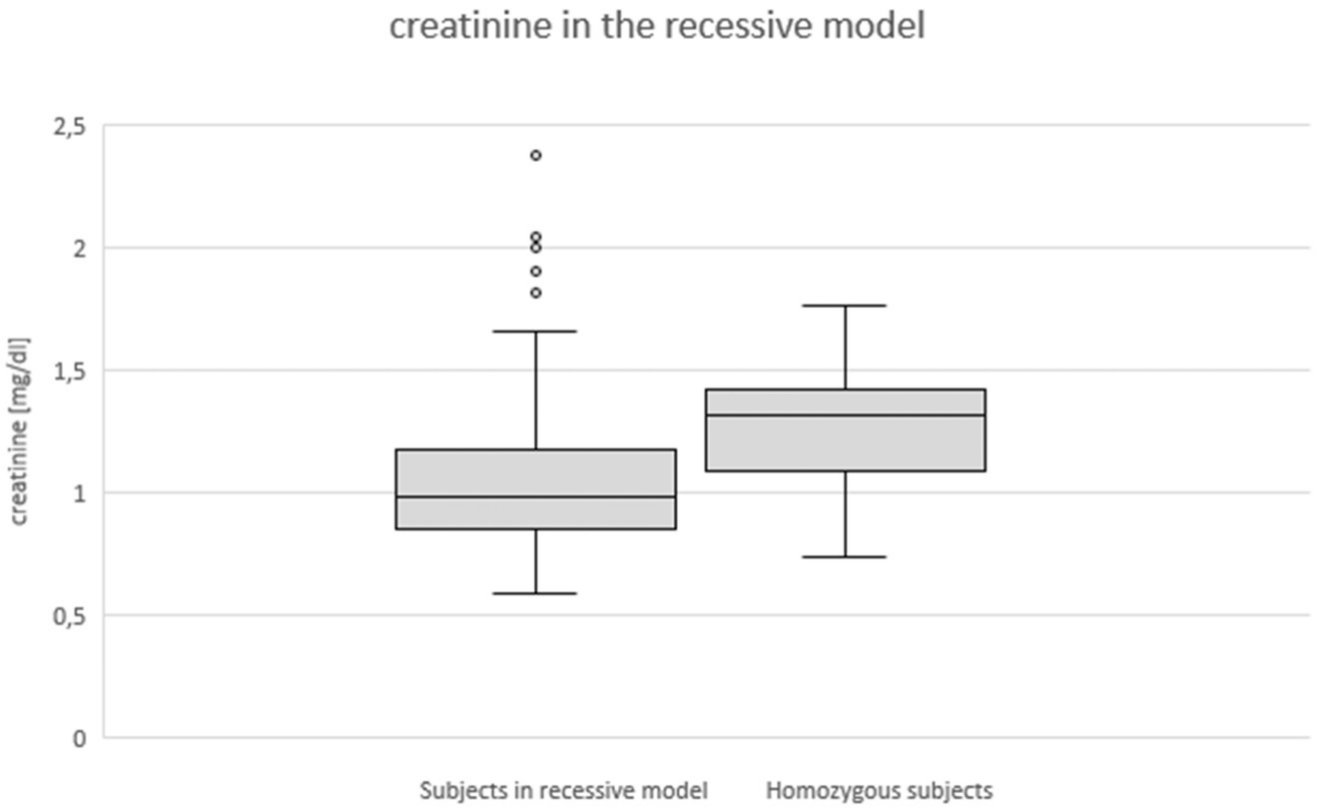
Genotype effect on serum creatinine in recessive model (p < 0.05).

**Table 1:** Clinical characteristics of patients according to the Leu33Pro polymorphism; values as mean ± SD or median with interquartile difference.

Factor	Wild-type (*n*=131)	Heterozygous (*n*=49)	Homozygous (*n*=6)
**Age (years)**	65 (10)	62 (16)	70 (40)
**BMI (kg/m^2^)**	29.4 (6)	30.0 (7)	30 (11)
**Cholesterol Total (mg/dL)**	193.1 ± 41	191.6 ± 31	181.8 ± 35
**HDL (mg/dl)**	46.7 (21)	46.2 (12)	46.8 (32)
**LDL (mg/dl)**	119.8 ± 35	191.6 ± 31	181.8 ± 35
**Triacylglycerol (mg/dL)**	175 (114)	167 (149)	118 (59)
**Fasting plasma glucose (mg/dL)**	153 (55)	130 (81)	157 (172)
**HbA1c (%)**	7 (2)	7.2 (2)	7.5 (2)
**CRP (mg/dL)**	0.95 (3)	0.28 (2)	0.41 (0)
**Creatinine (mg/dL)**	0.97 (0)	1 (0)	1.32 (1)
**GPT (U/L)**	26.5 (18)	28 (18)	18.5 (9)
**GGT (U/L)**	35 (31)	27 (28)	32 (49)
**Uric acid (mg/dL)**	5.9 (2)	5.7 (2)	8.3 (4)
**RR (mmHg)**	138 (26) / 80 (18)	139 (18) / 80 (4)	133 (43) / 82 (18)
**GFR (ml/min/1,73m^2^)**	73.4 ± 21	81.7 ± 22	62.3 ± 25

## Abbreviations

SNP: single nucleotide polymorphism
BMI: body mass index
HDL: high density lipoprotein
LDL: low density lipoprotein
SNP: single nucleotide polymorphism
GFR: glomerular infiltration rate
LPS: lipopolysaccharide
PMA: phorbol-12-myristat-13-acetat
RGD: tripeptide arginine, glycine, and aspartate

## References

[1] Schneider JG, Schiekofer S, Dugi KA (2009). The proline 12 alanine substitution in the ppargamma2 gene is associated with increased extent of coronary artery disease in men. Exp Clin Endocrinol Diabetes.

[2] Hynes RO (2002). Integrins: Bidirectional, allosteric signaling machines. Cell.

[3] Schneider JG, Zhu Y, Semenkovich CF (2007). Macrophage beta3 integrin suppresses hyperlipidemia-induced inflammation by modulating tnfalpha expression. Arterioscler Thromb Vasc Biol.

[4] Su X, Esser AK, Roomp K (2016). Antagonizing integrin β3 increases immunosuppression in cancer. Cancer Res.

[5] Reynolds LE, Wyder L, Hodivala-Dilke KM (2002). Enhanced pathological angiogenesis in mice lacking beta3 integrin or beta3 and beta5 integrins. Nat Med.

[6] Tschoepe D, Menart B, Roesen P (2003). Genetic variation of the platelet- surface integrin gpIIib-IIIa (pia1/a2-snp) shows a high association with type 2 diabetes mellitus. Diabetologia.

[7] Stratmann B, Xu T, Illig T (2014). Pla1a2 platelet polymorphism predicts mortality in prediabetic subjects of the population based kora s4-cohort. Cardiovascular diabetology.

[8] Jallu V, Poulain P, de Brevern AG (2014). Modeling and molecular dynamics simulations of the v33 variant of the integrin subunit β3: Structural comparison with the l33 (hpa-1a) and p33 (hpa-1b) variants. Biochimie.

[9] Rzezniczek S, Obuchowicz M, Pilc A (2016). Decreased sensitivity to paroxetine-induced inhibition of peripheral blood mononuclear cell growth in depressed and antidepressant treatment-resistant patients. Translational psychiatry.

[10] Verdoia M, Secco GG, De Luca G (2014). Platelet hpa-1 a/hpa-1 b polymorphism and the risk of periprocedural myocardial infarction in patients undergoing elective pci. Platelets.

[11] Maile LA, Busby WH, Patel A (2014). Blocking ligand occupancy of the αvβ3 integrin inhibits the development of nephropathy in diabetic pigs. Endocrinology.

[12] Zhao H, Ross FP, Teitelbaum SL (2005). Unoccupied alpha(v)beta3 integrin regulates osteoclast apoptosis by transmitting a positive death signal. Mol Endocrinol.

[13] Antonov AS, Antonova GN, Verin AD (2010). Alphavbeta3 integrin regulates macrophage inflammatory responses via pi3-kinase/akt-dependent nf-kappab activation. J Cell Physiol.

[14] Lim J, Lal S, Heng CK (2003). Variation of the platelet glycoprotein iiia pi(a1/a2) allele frequencies in the three ethnic groups of singapore. International journal of cardiology.

[15] Jin DK, Fish AJ, Kim Y (1996). Distribution of integrin subunits in human diabetic kidneys. Journal of the American Society of Nephrology.

[16] Staeck O, Slowinski T, Khadzhynov D (2015). Recurrent primary focal segmental glomerulosclerosis managed with intensified plasma exchange and concomitant monitoring of soluble urokinase-type plasminogen activator receptor-mediated podocyte β3-integrin activation. Transplantation.

